# A Descriptive Study on Multiple Health-Risk Behaviors among Chinese Adults in Hong Kong

**DOI:** 10.3390/ijerph191811393

**Published:** 2022-09-10

**Authors:** Ho Cheung William Li, Laurie Long Kwan Ho, Oi Kwan Joyce Chung, Ankie Tan Cheung, Wei Xia, Peige Song

**Affiliations:** 1 Nethersole School of Nursing, The Chinese University of Hong Kong, Hong Kong SAR, China; 2 School of Nursing, The Hong Kong Polytechnic University, Hong Kong SAR, China; 3 School of Nursing, Sun Yat-Sen University, Guangzhou 510275, China; 4 School of Public Health, Zhejiang University School of Medicine, Hangzhou 310027, China; 5 Centre for Global Health Research, Usher Institute of Population Health Sciences and Informatics, The University of Edinburgh, Edinburgh EH8 9YL, UK

**Keywords:** drinking alcohol, multiple health-risk behaviors, non-communicable diseases, smoking, physical inactivity, unhealthy diet

## Abstract

This study investigated the prevalence and clustering patterns of multiple health-risk behaviors and their associations with non-communicable diseases among Chinese adults in Hong Kong. A large sample survey was conducted in all 18 districts of Hong Kong between 21 June and 31 August 2021. A total of 5737 adults completed the survey. Overall, 4605 (80.3%) had at least one health-risk behavior and 2696 (47.0%) had two or more health-risk behaviors. Multiple health-risk behaviors were more prevalent among men. The prevalence of smoking and alcohol consumption among Hong Kong Chinese women was considerably lower than in most Western countries. In contrast to previous findings, this study revealed that a high proportion of adults with high educational attainment or household income had multiple health-risk behaviors. In addition, this study revealed that the health-risk behaviors in Chinese adults co-occurred in clusters, with smoking and alcohol consumption co-occurring with other health-risk behaviors. Those who smoked or consumed alcohol had the highest proportion of multiple health-risk behaviors and the highest proportion of non-communicable diseases. The findings of this study add further evidence that health-risk behaviors co-occur in clusters and can contribute to non-communicable diseases.

## 1. Introduction

The World Health Organization (WHO) has described non-communicable diseases (NCDs), including cardiovascular diseases, cancer, diabetes and chronic respiratory diseases, as the most common and preventable causes of morbidity and mortality worldwide [1,2]. According to the WHO, 41 million (71%) of the 57.7 million global deaths each year are attributed to NCDs [3]. The total number of annual deaths related to NCDs is expected to further increase to 55 million by 2030 unless urgent preventive measures are taken [1].

Hong Kong is facing an increasing burden of NCDs, which is compounded by population ageing [4]. In 2016, 25,771 registered deaths (approximately 55% of all deaths) were attributed to NCDs [5]. In addition, NCDs caused 104,600 potential years of life lost before the age of 70 [4].

The WHO has identified four major behavioral risk factors that substantially contribute to NCDs and can increase the risk of death; these are tobacco use, excessive alcohol use, an unhealthy diet and physical inactivity [1]. The WHO has also designated four metabolic risk factors: high blood pressure, overweight/obesity, hyperglycemia and hyperlipidemia [3]. Most premature deaths from NCDs could have been prevented through lifestyle modifications [6]. Therefore, assisting people to engage in healthy lifestyle practices, such as quitting smoking, avoiding excessive alcohol use, maintaining a balanced diet and engaging in regular physical activity, can help prevent NCDs and improve the overall health of the population. However, despite their awareness of the associated health hazards, many people lack motivation or find it difficult to modify their health-risk behaviors, especially when there is little advice and support from healthcare professionals [7,8,9,10]. In addition, health-risk behaviors co-occur in clusters, such that many people engage in multiple behaviors [11,12,13]. A sample of 16,818 adults from the 1998 US National Health Interview Survey revealed that 52% had two or more health-risk factors, including physical inactivity, overweight, cigarette smoking and excessive alcohol consumption [12]. Our previous study on youth smokers in Hong Kong found associations between smoking and physical inactivity, and between an unhealthy diet and alcohol consumption [14]. Research findings indicate that people with multiple health-risk behaviors have higher morbidity and mortality rates than those with a single health-risk behavior [11,12,13,14,15]. However, previous studies have shown that people with multiple health-risk behaviors face more challenges in the adoption of a healthy lifestyle than those with a single health-risk behavior [11,12,16]. Moreover, a review of the literature showed that many intervention studies of health-risk behaviors have targeted only a single behavior, while few have addressed multiple behaviors [7,8,9,10,12]. It is therefore crucial to develop and evaluate appropriate interventions that target people with multiple risk behaviors to help them refrain from engaging in health-risk behaviors and adopt a healthy lifestyle, either sequentially or concurrently. First, however, a thorough understanding of the clustering of multiple health-risk behaviors and the factors associated with such behaviors is crucial before appropriate interventions can be developed and evaluated. A review of the literature revealed that clustering patterns of multiple health-risk behaviors among the Chinese population are underexplored. Thus, this study aimed to investigate the prevalence and clustering patterns of multiple health-risk behaviors among Chinese adults in Hong Kong and their association with NCDs. 

## 2. Materials and Methods

### 2.1. Design and Sample

A transversal study was conducted. We surveyed a large sample to develop a robust understanding of multiple health-risk behaviors among Hong Kong Chinese adults. A convenience sample of Chinese adults aged 30 years or older who were able to speak Cantonese and read Chinese were recruited in the community. Hong Kong is a city divided into 18 districts. In order to enhance the representativeness of the sample and for ease of financial and manpower planning, we aimed to recruit participants from all the 18 districts evenly with roughly the same number of participants of at least 300 eligible adults. After receiving an explanation of the study’s purpose, informed written consent was obtained from each eligible participant. Data collection was conducted between 21 June and 31 August 2021, and 70 sessions of recruitment and survey activities were organized in 18 districts of Hong Kong. The survey was conducted from one district to another via face-to-face interviews by trained research assistants in pedestrian areas.

The reason for including people aged 30 years or older was that NCDs are increasingly seen in the younger population, although the majority of NCD onset occurs in those aged 40–50 years. 

### 2.2. Measures

A behavioral health-risk factor survey was used to collect demographic data and information regarding health-risk behaviors. The survey was a checklist adopted from the Hong Kong Department of Health (www.chp.gov.hk/files/pdf/brfs_2015apr_en.pdf, accessed on 5 January 2021). The Hong Kong Department of Health conducted a pilot study consisting of 52 successfully completed interviews before the population survey to test the length, logic, wording and format of the questionnaire. The demographic data included age, gender, socioeconomic status and presence of NCDs. Body mass index (BMI) and information on four major health-risk behaviors—(1) smoking, (2) alcohol consumption, (3) physical inactivity and (4) unhealthy diet—were collected. 

To assess smoking, participants were asked about their smoking history (currently, formerly or never). Current smokers were asked how many cigarettes they smoked on average per day and whether they had smoked at least one cigarette per day over the past 30 days. Alcohol consumption was assessed based on the frequency and amount of consumption and on the number of binge drinking episodes (the consumption of five or more standard drinks on one occasion) in the past year. To assess physical activity levels, participants were provided with examples of moderate and vigorous physical activities. They were then asked whether they had performed any moderate-intensity or vigorous-intensity aerobic physical activity in the previous 7 days and the duration of the activity. To assess diet, participants were asked on how many days of the previous 7 had they consumed fruit and vegetables and the amount consumed. Using fruit and vegetable consumption as a proxy of unhealthy diet is recommended by the Department of Health in Hong Kong and the World Health Organization (https://www.who.int/news-room/fact-sheets/detail/healthy-diet, accessed on 5 January 2021), and has been reported in the previous literature [17,18]. The criteria for determining whether the person engaged in each health-risk behavior are listed in Table 1.

This survey study was approved by the institutional review board of the University of Hong Kong and the Hospital Authority of Hong Kong, West Cluster (Reference number: UW 21-440). This study conformed to the principles embodied in the Declaration of Helsinki. Participants were given an information sheet that fully described the study’s purpose and nature. The participants were told that all questions being asked were related to their health and that the information provided by them would be kept strictly confidential and for collective analysis only. The participants could terminate the interview at any time without negative consequences. The survey took approximately 20 min to complete.

### 2.3. Data Analysis

SPSS for Windows (SPSS version 26.0; IBM Corp., Armonk, NY, USA) was used for the data analysis. Descriptive statistics were used to calculate the mean, standard deviation, and frequency of the demographic and health-risk behavior data. Demographic characteristics of participants with different health-risk behaviors were compared using the chi-square test (*χ*^2^). A multiple regression analysis was used to explore whether health-risk behaviors were associated with NCDs, while controlling for the possible effects of age, sex, household income and educational attainment.

## 3. Results

We approached 8687 Chinese adults and identified 7898 eligible participants during the data collection period. Overall, 2161 people refused to participate in the study because they were unable to stay for the 20 min required to complete the survey, while 5737 people participated in the study and completed the survey, giving a response rate of 72.6%. Table 2 presents the demographic data of the participants. There were similar numbers of men and women, with a mean participant age of 49.4 ± 13.1 years. The majority of participants (77%) were between 30 and 60 years old. Most participants were married (69.8%) and employed (67.1%); approximately half (44.4%) had completed upper secondary school education, and 32% had a household income above the median in Hong Kong (HKD 59,336 in 2021). According to the locally adapted classification of BMI for Chinese adults in Hong Kong, 54.6% of the respondents were classified as normal (BMI 18.5–23.0), 17.1% as overweight (BMI 23.0–25.0), 17.1% as obese (BMI > 25.0) and 11.2% as underweight (BMI < 18.5).

### Health-Risk Behaviors

Of the 5737 participants, 33.3%, 34.6%, 10.4% and 1.9% had one, two, three and four health-risk behaviors, respectively. Overall, 12.3% (704 of 5735) of the respondents were current smokers. About 12.8% (734 of 5737) of the participants consumed alcoholic beverages at least 1 day per week and 39.1% (287 of 734) reported that they had engaged in binge drinking (drinking five or more glasses/cans of alcohol on one occasion) in the past month. For physical activity, 75.5% (4330 of 5737) participants had not engaged in physical activity with at least 75 min of vigorous-intensity or 150 min of moderate-intensity aerobic physical activity per week as recommended by the WHO [19]. In addition, 2353 (41.0%) of the participants had consumed less than the WHO recommendation of five servings of fruit and vegetables per day or had a daily intake of less than 400 g of fruit and vegetables [20].

Significant statistically, more men (547/704) than women (157/704; χ^2^ = 187.7, *p* < 0.001) regularly smoked. Moreover, of statistical significance, more men (578/734) than women (156/734; χ^2^ = 215.1, *p* < 0.001) consumed alcohol.

Table 3 compares the demographic characteristics of participants with different numbers of health-risk behaviors. There were significant differences regarding gender, with more women having only one health-risk behavior (56.1%, 1070 of 1909) and more men having two or more health-risk behaviors. In particular, of the participants with three or four health-risk behaviors, 78.9% (472 of 598) and 92.8% (103 of 111) were men, respectively. Moreover, of those with three or four health-risk behaviors, a larger proportion were people with higher educational attainment or household income compared with those with lower educational attainment or household income. Additionally, a higher proportion of participants with four health-risk behaviors had NCDs (57.7%, 64 of 111) compared with those with only one health-risk behavior (28.2%, 537 of 1907).

Table 4 shows the clustering of four health-risk behaviors. Among all health-risk behaviors, smokers had the highest proportion of these with multiple health-risk behaviors (97.6%, 687 of 704). Those who regularly consumed alcohol had the second-highest proportion of multiple health-risk behaviors, with 94.7% (695 of 734) having more than one health-risk behavior.

The results also revealed clustering patterns of multiple health-risk behaviors. Among the 704 smokers, 231 (32.8%) regularly consumed alcohol; 632 (89.8%) were physically inactive and 425 (60.4%) had an unhealthy diet. Furthermore, 734 regularly consumed alcohol; 231 (31.5%) were smokers; 633 (86.2%) were physically inactive and 379 (51.6%) had an unhealthy diet. In addition, of the 4330 respondents who were physically inactive, 2147 (49.6%) had an unhealthy diet. 

Table 5 shows the summary results of a multiple regression analysis. The results showed that the overall model explained 39% of the variance. After controlling for the possible effects of age, sex, household income and educational attainment, we found that R2 change value was 0.28; that is, smoking, alcohol consumption, unhealthy diet, physical inactivity and number of health-risk behaviors explained an additional 28% of the variance in NCDs. When all variables were entered into the model, three variables (smoking, alcohol consumption and number of health-risk behaviors) made a statistically significant contribution (*p* < 0.05), which suggested that these factors were associated with NCDs. Additionally, the *β* coefficients for smoking, alcohol consumption and number of health-risk behaviors were 0.53, 0.26 and 0.36, respectively, indicating that smoking was a comparatively strong factor associated with NCDs.

## 4. Discussion

To the best of our knowledge, this is the first large survey study conducted to examine the number, types and clustering of health-risk behaviors among Hong Kong Chinese adults. A strength of this study is that the data collection was conducted through face-to-face interviews. Compared with large survey studies conducted via phone interviews, face-to-face interviews may enhance the response rate and the reliability of the findings [21]. Another strength is that this study was conducted in all 18 districts of Hong Kong and included a wide age spectrum of the population, thereby increasing the sample representativeness. Thus, the findings are more generalizable to the entire population. Consistent with the National Health Interview Survey conducted in the United States [12], this study showed that over 80% of participants (4605 of 5737) had at least one health-risk behavior, and 47.0% (2696 of 5737) had two or more health-risk behaviors. Physical inactivity was the most commonly reported health-risk behavior (75.5%). Physical inactivity has been identified as one of the behavioral risk factors that contribute to NCDs and is the major cause of premature death worldwide [22]. Although the Hong Kong government has made efforts to promote regular physical activity in the past few decades through health education in schools and social media campaigns, these findings indicate that the government should make greater efforts and incorporate new strategies to advocate that the public be physically active. Similar to the findings of previous studies [23,24], this study found that multiple health-risk behaviors were more prevalent among men. This study found that the proportion of Hong Kong Chinese women who regularly smoke or consume alcohol was considerably lower than in most Western countries [25,26]. Unlike in Western culture, in Chinese culture, it is less acceptable for women to smoke and consume alcohol [25,26]. In Hong Kong, some people still believe that only sex workers or ‘bad’ women smoke and consume alcohol; thus, there is a stigma associated with women who smoke or consume alcohol, who can be perceived as ‘bad’ or ‘evil’. Previous studies conducted in Western countries have found that multiple health-risk factors are more prevalent among those of lower social class, the economically inactive and those with less education [12,23,24]. In contrast to previous findings, this study revealed that a high proportion of adults with higher educational attainment or household income had multiple health-risk behaviors. One possible reason for this is that most middle-class people in Hong Kong are highly educated, white-collar professionals, but they are often referred to as the ‘sandwich class’ because of insufficient government support and inadequate social welfare, particularly a lack of government-subsidized public housing, which has placed increasing pressure on this population [27,28]. Moreover, many highly educated middle-class people work long hours, and their time is occupied by work and further education [29]. Consequently, they may encounter more difficulties, negative emotions and stress when trying to balance their busy family and working lives, and in paying for the soaring cost of private housing [26]. These difficulties may explain why they have a higher proportion of physical inactivity compared with other population groups. In addition, owing to constantly stressful life situations, they tend to engage in health-risk behaviors to relax, such as smoking and consuming alcohol [30]. Therefore, more attention should be given to this population. Specifically, healthcare professionals must focus on helping people understand the negative health consequences of health-risk behaviors and counsel them about alternative strategies for coping with negative emotions and stress. This study examined the clustering patterns of multiple health-risk behaviors among Hong Kong Chinese adults. In line with previous studies conducted in the West [11,12,13], health-risk behaviors in Chinese adults co-occurred in clusters. Of the 4605 participants identified to have at least one health-risk behavior (Table 3), more than half (58.5%, 2696 of 4605) had two or more health-risk behaviors. Similar to previous findings [11,31], this study identified clustering between smoking and alcohol consumption, and between an unhealthy diet and physical inactivity. This study revealed that people who smoked or consumed alcohol were more likely to be physically inactive and have an unhealthy diet. Previous studies have shown that cigarette smoking and alcohol consumption were associated with NCDS and could result in negative and serious health consequences [15,32]. This study adds further evidence to the literature that people with health-risk behaviors, especially those who regularly smoked, consumed alcohol and had multiple health-risk behaviors had a greater likelihood of having NCDs. Therefore, more attention, resources and effort should be given to help this vulnerable group by developing and evaluating effective interventions to help them adopt a healthy lifestyle. The results of this study indicate that multiple health-risk behaviors might contribute to the high morbidity and mortality rates associated with NCDs, which have a large impact not only on the health of individuals but also on families, the healthcare system, society and the economy [33]. Therefore, there is a need to adopt innovative approaches to motivate people to modify their health-risk behaviors and lead healthier lives. The implementation of targeted NCD interventions through the primary healthcare system is expected to improve the early screening, detection and timely treatment of NCDs. The early provision of interventions would help reduce the need for subsequent expensive treatments, thereby reducing healthcare expenditure and decreasing the economic burden on society. The successful prevention and control of NCDs relies on a collaborative effort by various stakeholders, including government bureaus and departments, academic and non-governmental organizations, the private sector and individuals. Healthcare professionals should work together to develop policies, systems, programs and actions to mitigate NCDs by strengthening health advocacy efforts, fostering partnerships to create supportive environments and enhancing NCD surveillance and progress monitoring. By conducting these activities, it is anticipated that in the long term there will be a meaningful and significant reduction in people’s risk of developing NCDs and in the morbidity and mortality rates associated with NCDs. This study has some limitations. First, this survey study relied on self-reported data. Hence, there might be response bias due to respondents under-reporting behaviors that are socially undesirable or over-reporting behaviors that are considered desirable. Second, there was a lack of objective assessment for NCDs. For example, some participants might not have been aware of having high blood pressure, glucose or cholesterol levels because the signs and symptoms for these metabolic risk factors are not obvious in the early stages. Consequently, self-reported NCDs might have been underestimated. To better quantify the impacts of health-risk behaviors on health outcomes, future studies should consider including biometric screening for NCDs, such as checking the participants’ blood pressure, performing a lung function test, and hyperglycemia and hyperlipidemia blood testing. Third, qualitative information, which might enable us to better understand participants’ perceptions, behaviors and attitudes related to their health-risk behaviors, was lacking. Finally, the transversal nature of this study limited the ability to verify causality of variables. Longitudinal studies are recommended for future studies to better understand how multiple risk factors contribute to NCD. Most importantly, in addition to measuring physical outcomes, participants’ psychological outcomes such as depressive symptoms, sleep patterns and the quality of life of participants should be assessed in future longitudinal studies.

## 5. Conclusions

This study filled a knowledge gap by investigating the prevalence and clustering patterns of multiple health-risk behaviors and their associations with NCDs among Hong Kong Chinese adults. The results revealed that 47.0% had multiple health-risk behaviors. Health-risk behaviors, especially smoking, alcohol consumption, and the presence of multiple health-risk behaviors, contribute to the development of NCDs and can increase the risk of death. Thus, the results of this study support the use of interventions to reduce these behaviors in the population. 

## Figures and Tables

**Table 1:** Criteria for determining engagement in health-risk behaviors.

Health-Risk Behavior	Criteria for Engaging in the Health-Risk Behavior
Tobacco use	Smoked at least one cigarette per day for the past 30 days
Alcohol consumption	Consumed alcohol regularly (i.e., at least 1 day per week) in the 12 months preceding the survey
Unhealthy diet	Consumed fewer than five servings of fruit and vegetables per day or had a daily intake of less than 400 g of fruit and vegetables
Physical inactivity	(1) Performed less than 150 min of moderate-intensity aerobic physical activity throughout the week, or (2) performed less than 75 min of vigorous-intensity aerobic physical activity throughout the week

**Table 2:** Demographic characteristics of the participants (*N* = 5737) ^a^.

Characteristic	Weighted Sample (%)
Age (Years)	
30 to 40	33.6
41 to 50	25.9
51 to 60	17.5
61–70	13.8
71–80	6.7
81–90	2.5
Sex	
Male	49.9
Female	50.1
Marital status	
Single	25.4
Married or cohabiting	69.8
Divorced, separated or widowed	4.8
Educational Attainment	
Primary school or below	11.2
Lower secondary school	14.7
Upper secondary school	44.4
Tertiary education	29.7
Household income	
HKD < 20,000	12.7
HKD 20,000–39,999	33.3
HKD 40,000–59,999	22.0
HKD 60,000 or above	32.0
Employment status	
Unemployed or retired	32.9
Employed	67.1
Body mass index classification	
Underweight (<18.5)	11.2
Normal (18.5 to 23.0)	54.6
Overweight (23.0 to 25.0)	17.1
Obese (>25)	17.1
Non-Communicable Diseases	
No	66.1%
Diabetes	10.6%
Chronic respiratory diseases	3.0%
Cancer	1.2%
Cardiovascular diseases	19.1%
Tobacco use	
Smokers	12.3%
Non-smokers	87.7%
Alcohol use	
Drinkers	12.8
Non-drinkers	87.2
Diet intake	
Unhealthy diet intake	41.0
Healthy diet intake	59.0
Physical activity	
No regular physical activity	75.5
Have regular physical activity	24.5

^a^ Sample sizes varied because of missing data on some variables.

**Table 3:** Demographic characteristics of the participants with different numbers of health-risk behaviors (*N* = 4605) ^a^.

	One Risk	Two Risks	Three Risks	Four Risks
Weighted Sample (%)				
WAge (Years)				
30 to 40	36.0	39.6	31.5	27.0
41 to 50	25.0	25.1	29.4	25.3
51 to 60	16.5	16.2	17.6	26.1
61–70	14.1	11.0	13.5	15.3
71–80	6.0	5.6	7.0	6.3
81–90	2.4	2.5	1.0	0.0
Sex				
Male	43.9	53.9	78.9	92.8
Female	56.1	46.1	21.1	7.2
Marital status				
Single	27.0	28.5	26.7	24.5
Married or cohabiting	69.5	65.9	67.5	66.7
Divorced, separated or widowed	3.5	5.6	5.8	8.8
Educational Attainment				
Primary school or below	11.4	10.2	9.7	12.0
Lower secondary school	12.9	12.7	20.8	23.1
Upper secondary school	49.2	45.2	33.4	26.9
Tertiary education	26.5	31.9	36.1	38.0
Household Income				
HKD < 20,000	27.2	2.6	0.8	0.0
HKD 20,000–39,999	28.9	35.6	21.2	19.8
HKD 40,000–59,999	23.6	24.1	39.3	30.7
HKD 60,000 or above	20.3	37.7	38.7	49.5
Employment status				
Unemployed or retired	30.9	29.6	25.4	27.9
Employed	69.1	70.4	74.6	72.1
BMI				
Underweight (<18.5)	12.7	11.4	7.7	11.6
Normal (18.5 to 23.0)	55.9	55.7	48.1	54.9
Overweight (23.0 to 25.0)	15.8	15.7	18.8	16.3
Obese (>25)	15.6	17.2	25.4	17.2
Non-Communicable Diseases				
No	71.8	66.5	55.2	42.3
Diabetes	9.2	11.0	14.5	13.6
Chronic respiratory diseases	1.9	3.1	6.2	9.0
Cancer	1.1	0.8	0.9	0.9
Cardiovascular diseases	16.0	18.6	23.2	34.2

^a^ Sample sizes varied because of missing data on some variables.

**Table 4:** The clustering of four health-risk behaviors (*N* = 4605).

	One Risk Behavior	Two Risk Behaviors	Three Risk Behaviors	Four Risk Behaviors
	Frequency (No./Total No. (%))			
Smoking	17/704 (2.4)	197/704 (28.0)	379/704 (53.8%)	111/704 (15.8)
Alcohol consumption	39/734 (5.3)	258/734 (35.1)	326/734 (44.4)	111/734 (15.1)
Unhealthy diet	141/2353 (6.0)	1584/2353 (67.3)	517/2353 (22.0)	111/2353 (4.7)
Physical inactivity	1712/4330 (39.5)	1935/4330 (44.7)	572/4330 (13.2)	111/4330 (2.6)
Total	1909/4605 (41.5)	1987/4605 (43.1)	598/4605 (13.0%)	111/4605 (2.4)

**Table 5:** Summary of multiple regression for variables predicting NCDs (*N* = 5737).

Variable Predicting NCDs	*B*	*SE B*	*β*	*p-*Value
Step 1				
Age	0.63	0.12	0.48	<0.001
Sex	−0.48	0.13	−0.43	<0.001
Household income	−0.37	0.08	−0.12	0.09
Educational attainment	−0.32	0.13	−0.13	0.17
Step 2				
Age	0.25	0.15	0.16	0.08
Sex	−0.32	0.13	−0.17	0.11
Household income	−0.09	0.03	−0.08	0.34
Educational attainment	−0.12	0.11	−0.09	0.52
Smoking	0.77	0.07	0.53	<0.001
Alcohol consumption	0.42	0.22	0.26	0.04
Unhealthy diet	0.09	0.09	0.17	0.33
Physical inactivity	0.12	0.11	0.20	0.24
Number of health-risk behaviors	0.55	0.12	0.36	0.02
R^2^ = 0.39				
Adjust R^2^ = 0.36				
R^2^ change = 0.28				

NCDs = non-communicable diseases; *B* = unstandardized coefficient; *SE B* = standard error of unstandardized coefficient; *β* = standardized coefficient.

## Data Availability

The data presented in this study are available on request from the corresponding author. The data are not publicly available due to privacy and ethical concerns.

## References

[1] (2013). World Health Organization, Global Action Plan for the Prevention and Control of Noncommunicable Diseases 2013–2020. https://www.who.int/publications/i/item/9789241506236.

[2] Elias M.A., Pati M.K., Aivalli P., Srinath B., Munegowda C., Shroff Z.C., Bigdeli M., Srinivas P.N. (2018). Preparedness for delivering non-communicable disease services in primary care: Access to medicines for diabetes and hypertension in a district in south India. BMJ Glob. Health.

[3] (2021). World Health Organization, Noncommunicable Diseases. https://www.who.int/news-room/fact-sheets/detail/noncommunicable-diseases.

[4] (2018). Hong Kong Food & Health Bureau, Towards 2025: Strategy and Action Plan to Prevent and Control Non-Communicable Diseases in Hong Kong. https://www.chp.gov.hk/files/pdf/saptowards2025_fullreport_en.pdf.

[5] (2016). Department of Health and Census and Statistics Department, 2016 Statistics of Inpatient Discharges and Deaths. https://www.ha.org.hk/haho/ho/stat/HASR16_17.pdf.

[6] GBD 2015 Risk Factors Collaborators (2016). Global, Regional, and National Comparative Risk Assessment of 79 Behavioural, Environmental and Occupational, and Metabolic risks or Clusters of risks, 1990–2015: A Systematic Analysis for the Global Burden of Disease Study 2015. Lancet.

[7] Li W.H.C., Ho K.Y., Xia V.W., Wang M.P., Lam K.K.W., Chan S.S.C., Lam T.H. (2019). Helping hospitalized smokers in Hong Kong quit smoking by understanding their risk perception, behaviour, and attitudes related to smoking. J. Adv. Nurs..

[8] Li H.C.W., Wang M.P., Ho K.Y., Lam K.K.W., Cheung D.Y.T., Cheung Y.T.Y., Lam T.H., Chan S.S.C. (2018). Helping cancer patients quit smoking using brief advice based on risk communication: A randomized controlled trial. Sci. Rep..

[9] Li H.C.W., Wang M.P., Lam T.H., Cheung Y.T.Y., Cheung D.Y.T., Suen Y.N., Ho K.Y., Tan K.C.B., Chan S.S.C. (2017). Brief intervention to promote smoking cessation and improve glycemic control in smokers with type 2 diabetes: A randomized controlled trial. Sci. Rep..

[10] Chan S.S.C., Leung D.Y.P., Wong D.C.N., Lau C.P., Wong V.T., Lam T.H. (2012). A randomized controlled trial of stage-matched intervention for smoking cessation in cardiac out-patients. Addiction.

[11] Geller K., Lippke S., Nigg C.R. (2017). Future directions of multiple behavior change research. J. Behav. Med..

[12] Coups E.J., Gaba A., Orleans T. (2004). Physician Screening for Multiple Behavioral Health Risk Factors. Am. J. Prev. Med..

[13] Spring B., Moller A.C., Coons M.J. (2012). Multiple health behaviours: Overview and implications. J. Public Health.

[14] Ho K.Y., Li H.C.W., Lam K.K.W., Chan S.S.C., Wang M.P., Chan V.W.F., Xia V.W., Lam T.H. (2018). Exploratory study on the relationship between smoking and other risk behaviours among young smokers. J. Clin. Nurs..

[15] Noble N., Paul C., Turon H., Oldmeadow C. (2015). Which modifiable health risk behaviours are related? A systematic review of the clustering of Smoking, Nutrition, Alcohol and Physical activity (“SNAP”) health risk factors. Prev. Med..

[16] Meader N., King K., Wright K., Graham H.M., Petticrew M., Power C., White M., Sowden A.J. (2017). Multiple risk behavior interventions: Meta-analyses of RCTs. Am. J. Prev. Med..

[17] (2010). World Health Organization, Global Recommendations on Physical Activity for Health. https://www.who.int/publications/i/item/9789241599979.

[18] McGuire S. (2013). State indicator report on fruits and vegetables, 2013, Centers for Disease Control and Prevention, Atlanta, GA. Adv. Nutr..

[19] Lam T.H., Chan B., Ho S.Y., Chan W.M. (2006). A prospective study of stage of change for general health promotion action and health-related lifestyle practices among Chinese adults. Soc. Sci. Med..

[20] World Health Organization (2003). Fruit, Vegetables and NCD Disease Prevention. http://who.int/dietphysicalactivity/media/en/gsfs_fv.pdf.

[21] Mahfoud Z., Ghandour L., Ghandour B., Mokdad A.H., Sibai A.M. (2015). Cell phone and face-to-face interview responses in population-based surveys: How do they compare?. Field Methods.

[22] World Health Organization (2019). Global Action Plan on Physical Activity 2018–2030: More Active People for a Healthier World. https://apps.who.int/iris/bitstream/handle/10665/272722/9789241514187-eng.pdf.

[23] Prochaska J.J., Velicer W.F., Nigg C.R., Prochaska J.O. (2008). Methods of quantifying change in multiple risk factor interventions. Prev. Med..

[24] Poortinga W. (2007). The prevalence and clustering of four major lifestyle risk factors in an English adult population. Prev. Med..

[25] Janghorbani M., Ho S.Y., Lam T.H., Janus E.D. (2003). Prevalence and correlates of alcohol use: A population-based study in Hong Kong. Addiction.

[26] Li H.C.W., Chan S.S.C., Lam T.H. (2015). Smoking among Hong Kong Chinese women: Behavior, attitudes and experience. BMC Public Health.

[27] Lee J. (1994). Affordability, home ownership and the middle class housing crisis in Hong Kong. Policy Polit..

[28] Chung R.Y., Mercer S., Lai F.T.T., Yip B.H., Wong M.C., Wong S.Y. (2015). Socioeconomic determinants of multimorbidity: A population-based household survey of Hong Kong Chinese. PLoS ONE.

[29] Stier H., Lewin-Epstein N. (2003). Time to work: A comparative analysis of preferences for working hours. Work Occup..

[30] Azagba S., Sharaf M.F. (2011). The effect of job stress on smoking and alcohol consumption. Health Econ. Rev..

[31] Meader N., King K., Moe-Byrne T., Wright K.l., Graham H., Petticrew M., Power C., White M., Sowden A.J. (2016). A systematic review on the clustering and co-occurrence of multiple risk behaviours. BMC Public Health.

[32] Holahan C.J., Holahan C.K., Lim S., Powers D.A. (2021). Living with a Smoker and Multiple Health-Risk Behaviors. Ann. Behav. Med..

[33] Murphy A., Palafox B., Walli-Attaei M., Powell-Jackson T., Rangarajan S., Alhabib K.F. (2020). The household economic burden of non-communicable diseases in 18 countries. BMJ Glob. Health.

